# 

**Published:** 1895-02

**Authors:** 


					﻿THE
Southern Medical Record.
A Monthly Journal of Medicine and Surgery.
foL. XXV.
ATLANTA, GA., FEBRUARY, 1895.
No. 2.
EDITORS:
1. W. GRIGGS, M. D.
r. McFadden gaston, m. d.
WILLIS F. WESTMORELAND, M. D.
LOUIS H. JONES, M. D.
DAN. H. HOWELL, M. D., Business Manager.
WM. S. GOLDSMITH, M. D., Managing Editor
TERMS: $2.00 Per Annum; Single Copy, 20 Cents.
Contents on Page 5.
PUBLISHED ON THE FIFTEENTH OF EACH MONTH.
Entered at the Post-office at Atlanta, Ga., as Second-Class Matter.
The' Foote & Davies Co., Atlanta, Ga.
				

